# p24 family proteins are critical for cell wall integrity, protein secretion, and virulence in *Candida albicans*

**DOI:** 10.1128/msphere.00827-25

**Published:** 2026-01-20

**Authors:** Xiangtai Yu, Hao Cui, Yifei Liu, Jian Yin, Jingkai Zhang, Gang Luo, Yang Lu, Chang Su

**Affiliations:** 1 Hubei Key Laboratory of Cell Homeostasis, College of Life Sciences, Wuhan University 98436, Wuhan, China; 2 State Key Laboratory of Metabolism and Regulation in Complex Organisms, College of Life Sciences, TaiKang Center for Life and Medical Sciences, Wuhan University 98436, Wuhan, China; University of Wisconsin-Madison, Madison, Wisconsin, USA

**Keywords:** *Candida albicans*, p24, protein secretion, virulence, cell wall integrity

## Abstract

*Candida albicans* is a fungal commensal and also a prevalent pathogen of humans. p24 proteins are a family of type I membrane proteins regarded as cargo receptors for endoplasmic reticulum (ER) to Golgi transport and are thought to be involved in regulating secretion. Here, we sought to explore the impact of this family of proteins on *C. albicans* pathogenicity. The expression of all four members of the p24 family is upregulated during invasive candidiasis. Their expression is independent of yeast-to-hypha transition but is highly induced by tissue culture conditions. We then generated single deletion mutants for each member of the p24 family for phenotypic characterization. All these mutants exhibit significantly attenuated virulence in a mouse model of systemic infection and reduced survival in macrophages but are dispensable for vegetative growth and morphogenesis. They also show lower abundance of chitin and phosphomannan in the cell wall and enhanced sensitivity to fluconazole, an azole antifungal drug. Importantly, the absence of p24 proteins leads to defective protein secretion in *C. albicans*, including pathogenicity-related effectors and lipases, and reduces commensal fitness. These results suggest that p24 proteins are critical for cell wall integrity, secretion of virulence factors, and virulence in *C. albicans*.

**IMPORTANCE:**

*Candida albicans* is an important opportunistic fungal pathogen of immunocompromised individuals and a top-ranking WHO fungal priority pathogen due to the high frequency and mortality of invasive candidiasis. The eukaryotic p24 family of proteins has long been known to be key regulators of protein trafficking along the secretory pathway, but their potential roles regarding pathogenesis in *C. albicans* remain unknown. Here, we discover that all members of the p24 family are required for cell wall integrity, proper secretion of virulence factors, survival in macrophages, and virulence in a systemic infection model. However, they are dispensable for vegetative growth and yeast-to-hypha transition, the best-known virulence attribute. Our study systematically investigates *C. albicans* p24 proteins and highlights the critical role that the early secretory pathway plays in fungal pathogenicity.

## INTRODUCTION

*Candida albicans* exists as a fungal commensal that stably colonizes mucosal surfaces and the gastrointestinal tract without causing pathology (1). However, this fungus is poised to cause serious invasive infections with high frequency and mortality when antifungal immune defenses are compromised (2, 3). *C. albicans* is classified in the World Health Organization critical priority group (4). Primary risk factors for invasive candidiasis include colonization, immunodeficiency, the presence of a central venous catheter, and microbial dysbiosis (3).

Proper protein secretion is critical for fungal development and pathogenesis (5–8). This is also the case for *C. albicans*. This fungus expresses a number of proteases, lipases, and effectors, which function in degrading host connective tissues, targeting host immune defense proteins, and thus aiding nutrition acquisition, invasion, and evasion of host immune defenses (9–12). It is widely believed that these virulence factors are delivered along the secretory pathway to their destinations. Despite significant advances in the secretory pathway in *C. albicans* (13–15), it remains incompletely understood.

Protein secretion in eukaryotes depends mainly on the conventional endoplasmic reticulum (ER)-to-Golgi secretory pathway (16–18). p24 proteins are a family of type I transmembrane proteins highly conserved among eukaryotes. They can be classified, by sequence homology, into four subfamilies: p24α, p24β, p24γ, and p24δ (19–21). p24 proteins are long known to cycle between the ER and the Golgi via coat protein I (COPI) and COPII vesicles for efficient protein sorting (20–23), a process involved in transport of cargo along the secretory pathway. p24 proteins have been shown to be essential for transport from the ER to the plasma membrane of glycosylphosphatidylinositol (GPI)-anchored proteins (24, 25). They have also been shown to be involved in the transport of Wnt proteins (26, 27), G-protein-coupled receptors (GPCRs) (28), plant myrosinase-associated protein GLL23 (29), insulin (30, 31), and Toll-like receptor 4 (32). Despite increasing interest in these proteins, very little is known about their functions in the pathogenic fungus *C. albicans*.

Here, we have carried out a systematic analysis of the p24 family in *C. albicans*. Phenotypic loss of function analysis of p24 proteins has revealed defects in cell wall integrity, virulence, and commensal fitness. In addition, p24 proteins are required for proper secretion of certain virulence factors. Thus, our study characterized the biological functions of the p24 family in *C. albicans* and provided a better understanding of the pathogenesis mechanisms of fungal pathogens.

## RESULTS

### Identification and characterization of p24 family proteins in *C. albicans*

**Fig 1:**
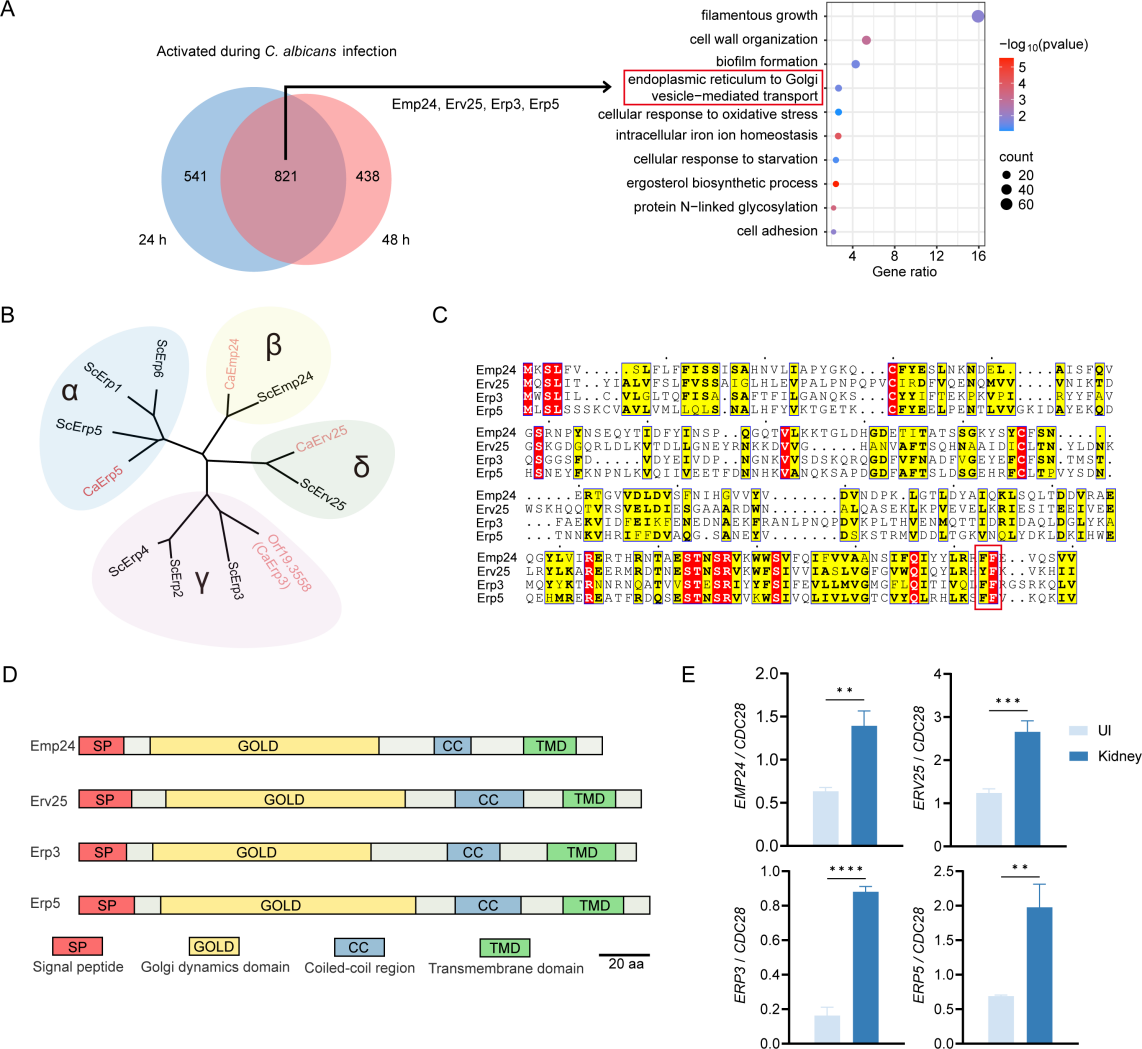
Transcriptional profiling of *in vivo* RNA-Seq and sequence analysis of p24 family members in *C. albicans*. (**A**) Venn diagram indicates a significant overlap of genes upregulated at 24 h with 48 h post-infection. Activation was defined by a minimum 1.5-fold increase of gene expression. The false discovery rate was filtered to 0.05. GO analysis of genes differentially expressed at both 24 h and 48 h during *C. albicans* infection was performed using DAVID (http://david.ncifcrf.gov). (**B**) Unrooted tree of the p24 protein family in *S. cerevisiae* and *C. albicans*. The tree was generated using the molecular evolutionary genetics analysis software (MEGA) with the Neighbor-Joining method (34). The four p24 subfamilies (α, β, γ, and δ) are highlighted by color background shading. (**C**) Alignment of p24 family proteins in *C. albicans*. The single-letter code for amino acids is used. The ΦF motif present in the cytosolic C-terminus of p24 family proteins is boxed in red. Φ is a bulky hydrophobic residue (F/Y/L/I). (**D**) The alignment of signal peptide, Golgi dynamics domain, coiled-coil region, and transmembrane domain in p24 proteins is shown. aa, amino acids. (**E**) qRT-PCR analysis for the expression of *EMP24*, *ERV25*, *ERP3,* and *ERP5* during invasive infection of *C. albicans*. WT *C. albicans* (SC5314) cells were grown in liquid YPD medium at 30°C for an *in vitro* control and then administered to 19–21 g male BABL/c mice by tail vein injection. At 48 h pi, total RNA of the infected kidneys was extracted. The signals obtained from *CDC28* mRNA were used for normalization. Error bars represent standard deviations from the means of three experiments. Significance was measured with an unpaired *t*-test in GraphPad Prism. **, *P* < 0.01; ***, *P* < 0.001; ****, *P* < 0.0001.

To explore the transcriptional response of *C. albicans* during invasive infection, we conducted *in vivo* RNA sequencing using a murine model with intravenous administration, as described in our recently published study (11). RNA-seq analysis was performed on kidneys, the most heavily infected organ, at 24 and 48 h post-infection (pi). *C. albicans* cells cultured in YPD medium at 30°C were used as a control to identify differentially expressed genes during *in vivo* infection. A total of 821 *C*. *albicans* genes were upregulated by at least 1.5-fold at both 24 and 48 h pi (Fig. 1A; Table S1). Gene Ontology (GO) analysis of these genes revealed significant enrichment in the category related to ER-Golgi vesicle-mediated transport (Fig. 1A). We chose *EMP24*, *ERV25*, *ERP3* (*ORF19.3558*), and *ERP5*, which encode the proteins of the p24 family, for further investigation, as they are crucial for protein secretion in eukaryotes (20, 33).

Based on sequence homology, p24 proteins are classified into four subfamilies designated α, β, δ, and γ (19). Within each of the four p24 subfamilies, amino acid sequence identity is remarkably high. In the model yeast *Saccharomyces cerevisiae*, the p24 family consists of eight members, with the p24α and p24γ subfamilies showing expansion, while the p24β and p24δ subfamilies each contain only a single member (Fig. 1B; Fig. S1A through D). An extensive search of the *C. albicans* genome database (http://www.candidagenome.org/) revealed four p24 family member genes—*EMP24*, *ERV25*, *ERP3* (*ORF19.3558*), and *ERP5*—distributed into four conserved subfamilies, respectively (Fig. 1B; Fig. S1A through D). *C. albicans* p24 proteins of all four subfamilies share extensive sequence similarity (Fig. 1C). They display a common modular structure consisting of a cleavable signal peptide, a lumenal part with a Golgi dynamics (GOLD) domain (35), a coiled-coil region involved in oligomerization (36, 37), and a single hydrophobic transmembrane region (Fig. 1D). Additionally, a short cytosolic tail that contains well-characterized COPI and COPII recruiting motifs (ΦF) responsible for their cycling between the ER and Golgi is present in all four proteins (19, 38). Φ represents a bulky hydrophobic residue (F/Y/L/I) (Fig. 1C). The upregulation of all four p24 family genes during invasive infection was validated by qPCR analysis (Fig. 1E).

To elucidate the functional role of the p24 family in *C. albicans*, null mutants for each gene in this family, in which the whole open reading frame was deleted, were constructed using CRISPR-Cas9 (39). The *emp24*Δ/Δ, *erv25*Δ/Δ, *erp3*Δ/Δ, and *erp5*Δ/Δ mutants and their corresponding complemented strains were successfully generated, as verified by PCR and sequencing (Fig. S2A and B). To assess the functional consequences of p24 family gene deletions, we first conducted phenotypic analyses focusing on growth characteristics and morphological development. Growth was evaluated in YPD liquid medium at 30°C over a 14-hour period. Notably, all mutant strains exhibited growth patterns that were indistinguishable from the WT strain (Fig. S3A). Similarly, these deletion strains demonstrated no discernible growth defects at either 30°C or 37°C on YPD solid medium (Fig. S3B). Hyphal morphogenesis, which represents a key virulence determinant in *C. albicans* (40, 41), was assessed using serum-containing medium and RPMI-1640 medium at 37°C. As shown in Fig. S2C, these mutants exhibited similar patterns of filamentation compared to the WT strain under these conditions, indicating that p24 proteins are dispensable for yeast-to-hypha transition. p24 proteins are critical for *C. albicans* virulence.

**Fig 2:**
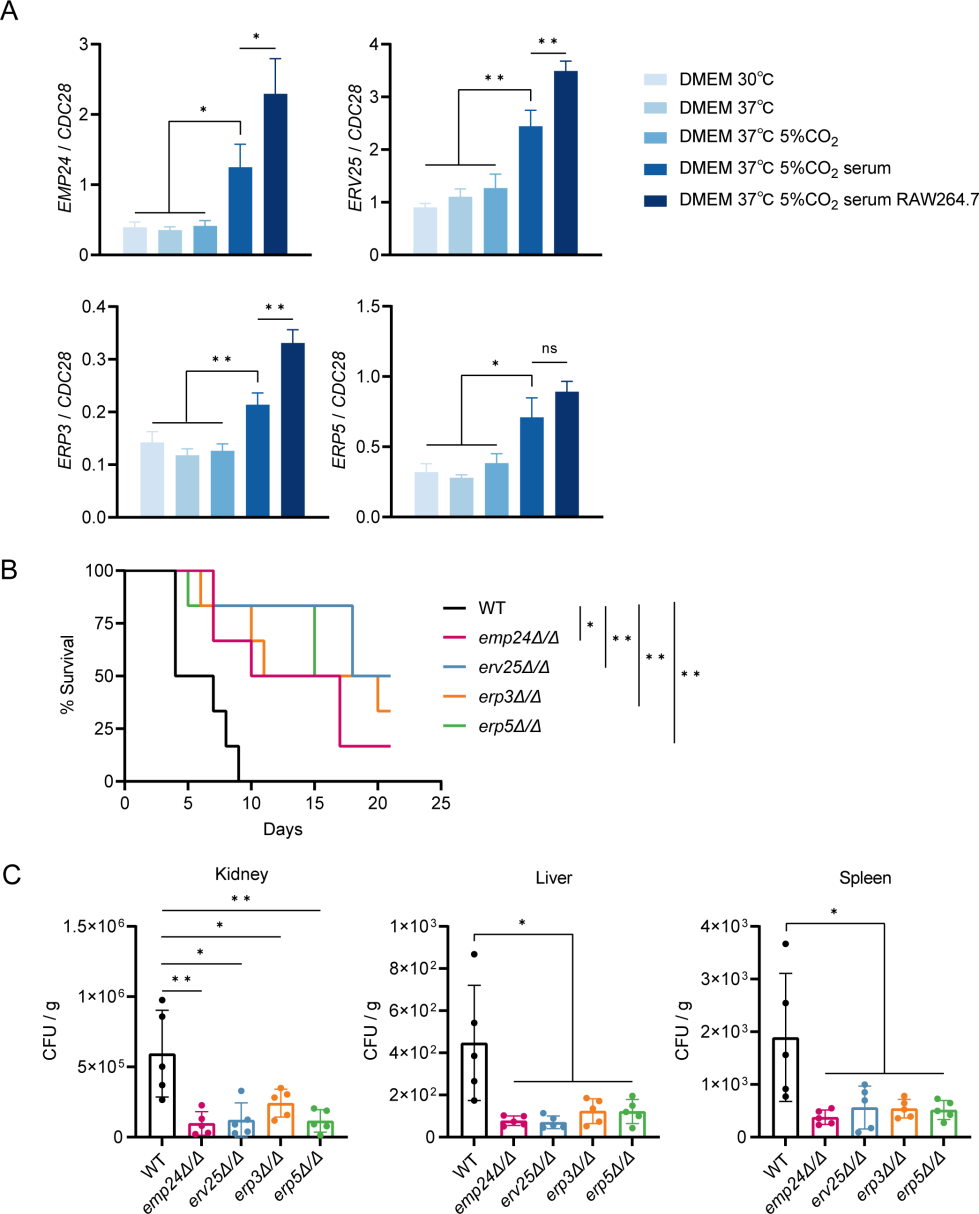
p24 family genes are induced by tissue culture conditions and are critical for virulence. (**A**) qRT-PCR analysis of *EMP24*, *ERV25*, *ERP3,* and *ERP5* mRNA in WT *C. albicans* cells incubated under indicated conditions. The signals obtained from *CDC28* mRNA were used for normalization. Error bars represent standard deviations from the means of three experiments. Significance was measured with an unpaired *t*-test in GraphPad Prism. (**B**) Mutant strains of *emp24*, *erv25*, *erp3,* and *erp5* exhibited reduced lethality compared with the WT strain. Groups of male BALB/c mice (19–21 g) were injected with 5 × 10^5^ CFU of *C. albicans*. Statistical significance was determined by log-rank test. *n* = 6 mice. (**C**) *emp24*, *erv25*, *erp3*, and *erp5* mutants failed to persist in infected organs. Groups of male BALB/c mice were infected with 2.5 × 10^5^ CFU of *C. albicans*, followed by euthanasia of five animals per group after 5 days. CFUs were determined by plating kidney, liver, or spleen homogenates onto agar plates supplemented with streptomycin and ampicillin and counting after growth for 2 days. Data represent the mean and standard deviations. Statistical significance was determined by an unpaired *t*-test. ns, no significance; *, *P* < 0.05; **, *P* < 0.01.

We next asked whether morphogenesis or culture conditions had any impact on the expression of p24 family genes. The expression of p24 family genes was not affected by yeast-to-hyphae transition, as exposure of WT *C. albicans* to hypha-promoting condition did not upregulate their expression (Fig. S4, RPMI-1640, 37°C vs YPD, 30°C). By contrast, we found that tissue culture conditions (37°C, DMEM, 10% fetal calf serum, 5% CO_2_) dramatically increased mRNA levels of p24 family genes (Fig. 2A). The expression levels of p24 family genes could be further enhanced by addition of murine macrophages (RAW264.7) to the tissue culture medium (Fig. 2A).

To investigate the role of p24 proteins in *C. albicans* pathogenesis, we assessed their impact on virulence using a murine model of systemic infection. WT and strains lacking p24 family genes were injected into mice via the tail vein, and survival was monitored over 21 days. All mice injected with WT strain died within 10 days (Fig. 2B; Fig. S5). In contrast, *emp24*Δ/Δ, *erv25*Δ/Δ, *erp3*Δ/Δ, and *erp5*Δ/Δ mutants demonstrated significantly attenuated virulence, as a considerable portion of mice infected with these mutant strains survived throughout 21 days (Fig. 2B). Complementation of these mutant strains with corresponding WT genes rescued their virulence defect (Fig. S5). Correspondingly, mice infected with the *emp24*Δ/Δ, *erv25*Δ/Δ, *erp3*Δ/Δ, and *erp5*Δ/Δ mutant strains exhibited significantly lower fungal loads in the kidneys, livers, and spleens compared to those infected with the WT strain (Fig. 2C). This is consistent with a previous study where the *emp24*Δ/Δ mutant showed attenuated virulence in screening competition assay (42). Collectively, our data reveal that p24 proteins are crucial for *C. albicans* pathogenicity.

### p24 proteins are critical for *C. albicans* survival in macrophages and stress response

**Fig 3:**
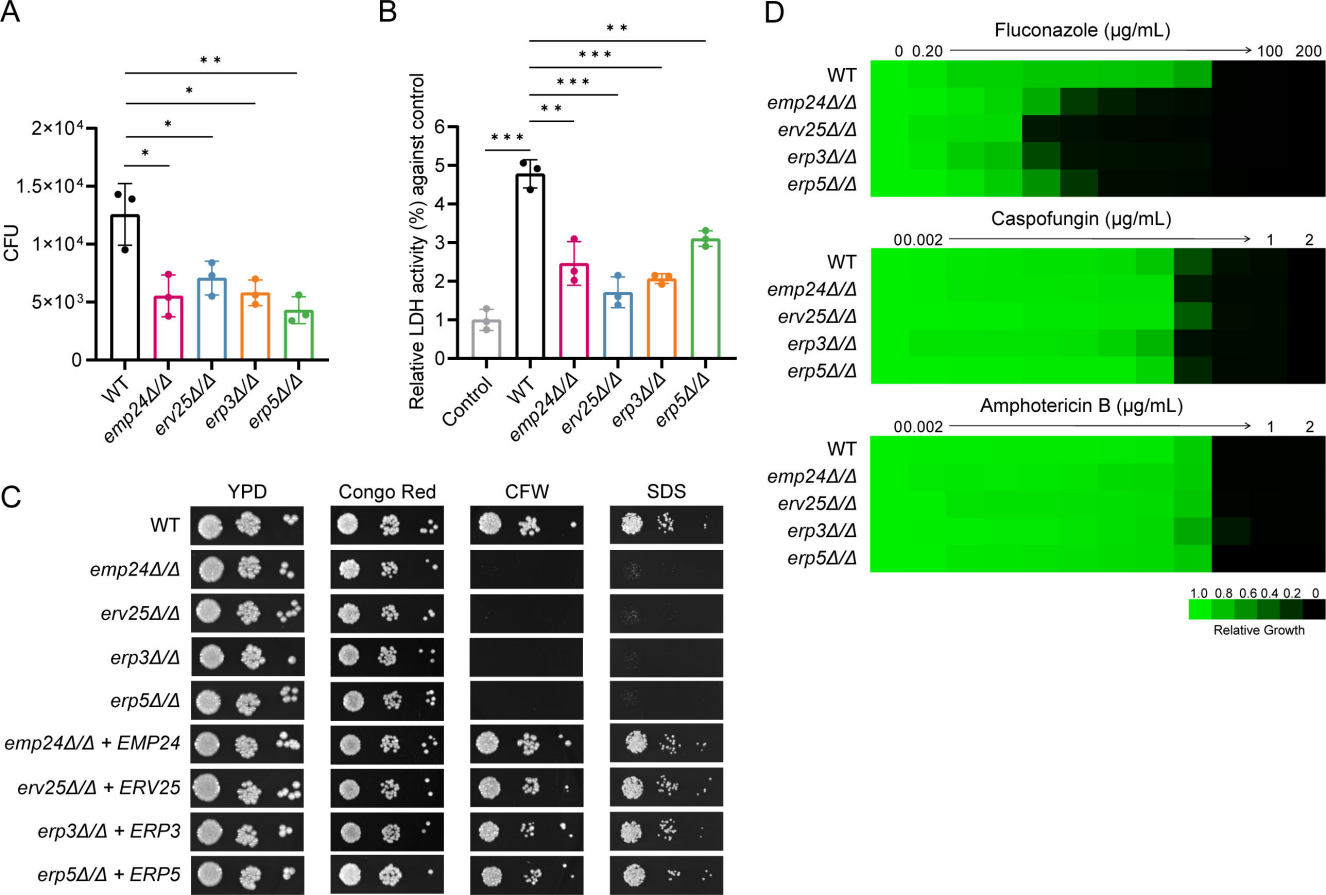
Loss of p24 family genes leads to reduced survival in macrophage and defective cell wall integrity. (**A**) RAW264.7 cells were cultured with the WT strain and indicated mutant strains. Non-phagocytosed *C. albicans* cells were removed by washing with PBS after 1 h, and the CFUs of *C. albicans* in RAW264.7 cells were determined after co-incubation for an additional 5 h. *n* = 3 biologically independent samples. (**B**) Cytotoxicity assays. RAW264.7 cell damage following treatment with the WT strain and indicated mutant strains was determined after co-incubation. Relative LDH activity (%) against RAW264.7 cells without *C. albicans* cells was calculated. Mean data ± SD from three independent experiments was plotted. (**C**) Wild-type *C. albicans*, the indicated deletion mutants, and their complemented derivatives were challenged with stress agents, including Congo red (50 µg/mL), CFW (35 µg/mL), and SDS (0.025%). Photographs were taken after growth at 30°C for 48 h. (**D**) Fluconazole, caspofungin, and amphotericin B susceptibility assays were conducted in YPD medium for the WT strain and indicated mutants. Growth was measured by absorbance at 600 nm after 48 h at 30°C. Optical densities were normalized relative to those of antifungal drug-free controls. Data are quantitatively displayed in heat map format (see color bar). (**A and B**) Statistical significance was determined by an unpaired *t* test. *, *P* < 0.05; **, *P* < 0.01; ***, *P* < 0.001.

We next examined the fungistatic activity of macrophages toward WT and p24 mutants. A significant attenuation of survival was observed in *emp24*Δ/Δ, *erv25*Δ/Δ, *erp3*Δ/Δ*,* and *erp5*Δ/Δ mutants compared with the WT strain after a 6-hour exposure to macrophages (Fig. 3A). Correspondingly, *emp24*Δ/Δ, *erv25*Δ/Δ, *erp3*Δ/Δ, and *erp5*Δ/Δ mutants killed far fewer macrophages than the WT strain, as indicated by the lower lactate dehydrogenase (LDH) release from macrophages when challenged with these mutant strains (Fig. 3B). Adding back WT genes into corresponding mutants restored these phenotypes (Fig. S6A and B). These findings indicate that p24 proteins are crucial for *C. albicans* to counteract macrophage killing.

We then asked how p24 proteins impact *C. albicans* survival within macrophages. We found that deletion of p24 family genes resulted in increased sensitivity to the cell wall-perturbing compound CFW that stains chitin and sodium dodecyl sulfate (SDS), which causes membrane perturbation (Fig. 3C). Complementation of the mutants with the corresponding WT genes rescued their growth defect (Fig. 3C). However, *emp24*Δ/Δ, *erv25*Δ/Δ, *erp3*Δ/Δ, and *erp5*Δ/Δ mutants grew normally under cell wall stress challenge with Congo red, under osmotic stress challenge with KCl, under oxidative stress challenge with H_2_O_2_, and at elevated temperature (42°C) (Fig. 3C; Fig. S7). We further demonstrated that deleting p24 family genes led to increased sensitivity toward fluconazole, but not caspofungin or amphotericin B (Fig. 3D). These data indicate that p24 proteins are critical for stress response and antifungal drug tolerance in *C. albicans*.

### Deletion of p24 family genes leads to alteration in cell wall structure

**Fig 4:**
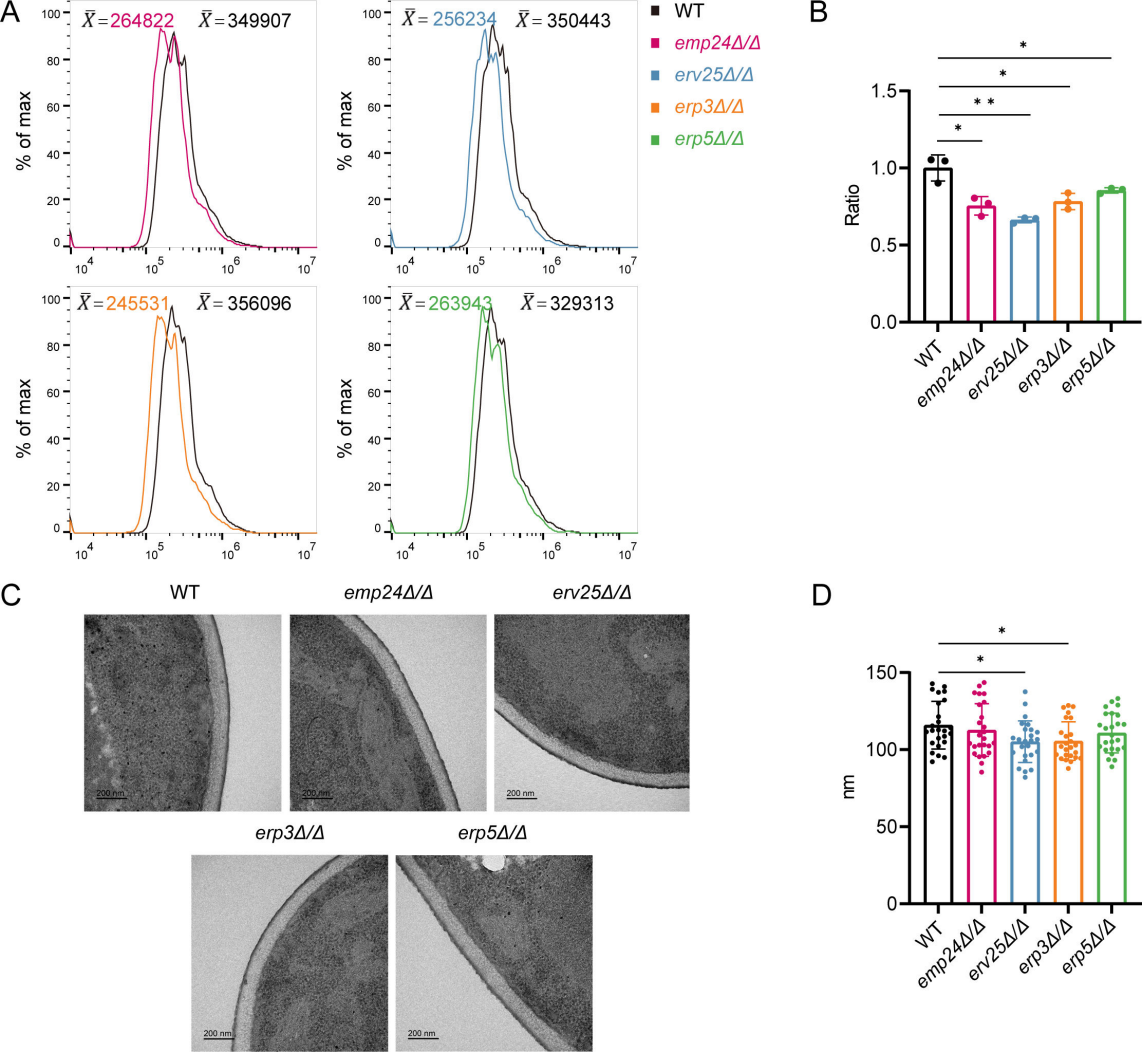
Loss of p24 proteins results in altered cell wall structure. (**A**) Flow cytometric analysis of cell wall chitin content in WT and indicated mutant strains. Log-phase *C. albicans* cells were stained with CFW (3.5 µg/mL) and subjected to fluorescence-activated cell sorting (FACS) analysis. Average fluorescence of the population was indicated. Plots are representative of data collected in three independent replicate experiments. (**B**) The incorporation of phosphomannan into the fungal cell wall was assessed using Alcian Blue staining. Optical density was measured by absorbance at 620 nm following staining with 30 µg/mL Alcian Blue, with values averaged from three independent experiments. (**C**) Transmission electron microscope (TEM) images of cell walls from *C. albicans* cells grown in liquid YPD medium at 30°C. Scale bar, 200 nm. (**D**) Quantification of cell wall thickness based on TEM pictures using ImageJ (*n* = 25 cells). (**B and D**) Significance was measured with an unpaired *t*-test in GraphPad Prism. *, *P* < 0.05; **, *P* < 0.01.

Given that p24 mutants exhibited increased sensitivity to CFW, we investigated whether chitin abundance was altered upon deletion of p24 family genes. The median fluorescence intensity of *Candida* cells was measured by flow cytometry following CFW staining. As shown in Fig. 4A, a reduction in the abundance of cell wall chitin is observed in *emp24*Δ/Δ, *erv25*Δ/Δ, *erp3*Δ/Δ, and *erp5*Δ/Δ mutants compared to the WT strain. In addition, we employed Aniline Blue and Concanavalin A (ConA) to measure glucan and mannan levels, two other major components of the cell wall. As shown in Fig. S8, deletion of p24 family genes did not alter glucan and mannan abundance in the cell wall. Since surface β-glucan masking plays an important role in immune evasion, we also examined surface-exposed β-glucan using Fc-dectin-1 and found that the surface-exposed β-glucan levels in all these mutant strains were comparable to that of WT strain (Fig. S8). In contrast, using Alcian Blue, a cationic dye that specifically binds phosphomannan groups, we detected a marked reduction in cell wall phosphomannan levels when p24 family genes were deleted (Fig. 4B). These findings demonstrate that p24 proteins are required for cell wall integrity.

We next investigated cell wall thickness in strains lacking p24 proteins. Cell wall thickness was measured in 25 individual cells per strain, and a representative cell wall image for each strain is shown in Fig. 4C. WT *C. albicans* exhibited an average cell wall thickness of 115.9 nm, as measured from the plasma membrane to the outer boundary of the cell wall. For *emp24*Δ/Δ, *erv25*Δ/Δ, *erp3*Δ/Δ, and *erp5*Δ/Δ mutant strains, the cell wall thickness was 105.6 nm, 110.8 nm, 112.7 nm, and 105.2 nm, respectively (Fig. 4D), indicating that deletion of p24 family genes resulted in a slight reduction in cell wall thickness. All these phenotypes regarding alterations in cell wall structure could be restored by adding back WT genes into the corresponding mutant strains (Fig. S9A through D). These results support a model in which p24 proteins have an impact on cell wall architecture.

### Loss of p24 proteins leads to defective intestinal colonization but does not affect mucosal infection

**Fig 5:**
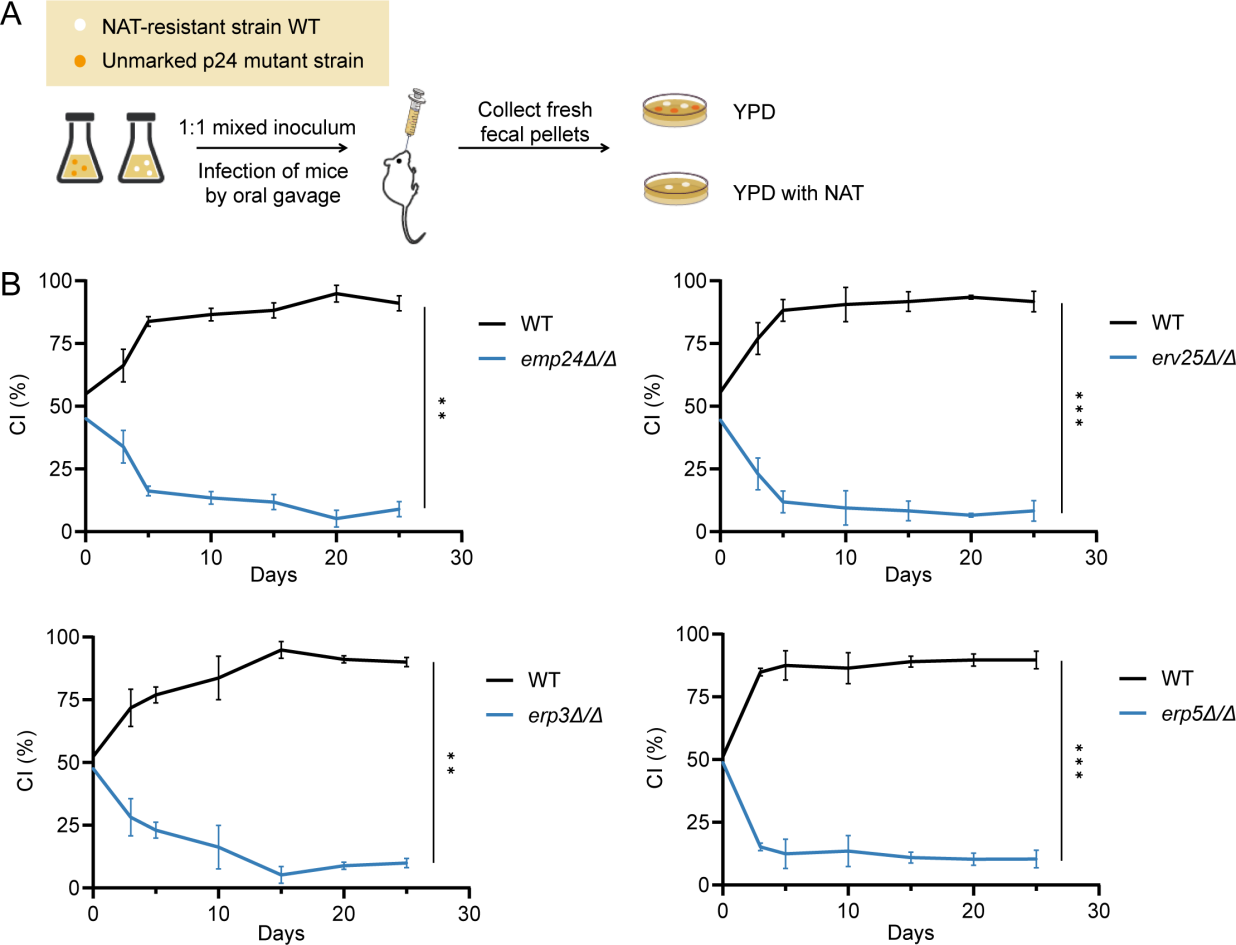
p24 proteins promote gut colonization of *C. albicans*. (**A**) Schematic of commensal competition experiments. 19-21 g female BALB/c mice were gavaged with 1:1 mixtures of a nourseothricin-resistant WT strain and an unmarked *emp24*Δ/Δ, *erv25*Δ/Δ, *erp3Δ/Δ*, or *erp5*Δ/Δ mutant strain. Relative abundance of strains was monitored over a time course of 25 days by collecting fresh fecal pellets and plating homogenates on YPD plates supplemented with or without 200 μg/mL nourseothricin. (**B**) Competition between the WT and *erv25* mutant strain. *n* = 3 mice. The competitive index (CI) was shown as the proportion of the indicated strain to the total. Data are presented as the mean ± SD. Significance was determined using the two-tailed Student’s *t*-test. **, *P* < 0.01; ***, *P* < 0.001; ****, *P* < 0.0001.

Given the critical role of cell wall in fungi-host interaction, we sought to explore the impact of p24 proteins on intestinal colonization of *C. albicans*. A competitive colonization assay was performed by orally inoculating mice with a 1:1 mixture of a nourseothricin (NAT)-resistant WT strain and an unmarked *ERV25* deletion strain, a member of the p24 family (Fig. 5A). The NAT marker was previously shown to have no impact on commensal fitness (43). At multiple time points post-inoculation, fecal samples were collected, homogenized, and plated to quantify CFU per gram (CFU/g) and to calculate the competitive index (CI), defined as the ratio of the indicated strain. As shown in Fig. 5B, *emp24*Δ/Δ, *erv25*Δ/Δ, *erp3*Δ/Δ, and *erp5*Δ/Δ mutants display significantly reduced commensal fitness compared to WT strain, indicating that the p24 proteins promote *C. albicans* gut colonization.

Having elucidated the critical role that p24 proteins play in both gut colonization and invasive infection of *C. albicans*, we then asked whether p24 proteins are important for mucosal infections. To test this, we compared fungal burdens in tongue tissues of mice infected with the WT strain, the *erv25*Δ/Δ mutant, and the complemented strain after a 2-day oropharyngeal infection using an established immunosuppressed mouse model (44). As shown in Fig. S10, no statistically significant differences in fungal burden are detected between the WT strain and *erv25* mutant in infected tongue tissues. This result indicates that p24 proteins have no impact on mucosal infections of *C. albicans*.

### p24 proteins are required for secretion of pathogenicity-related proteins in *C. albicans*

**Fig 6:**
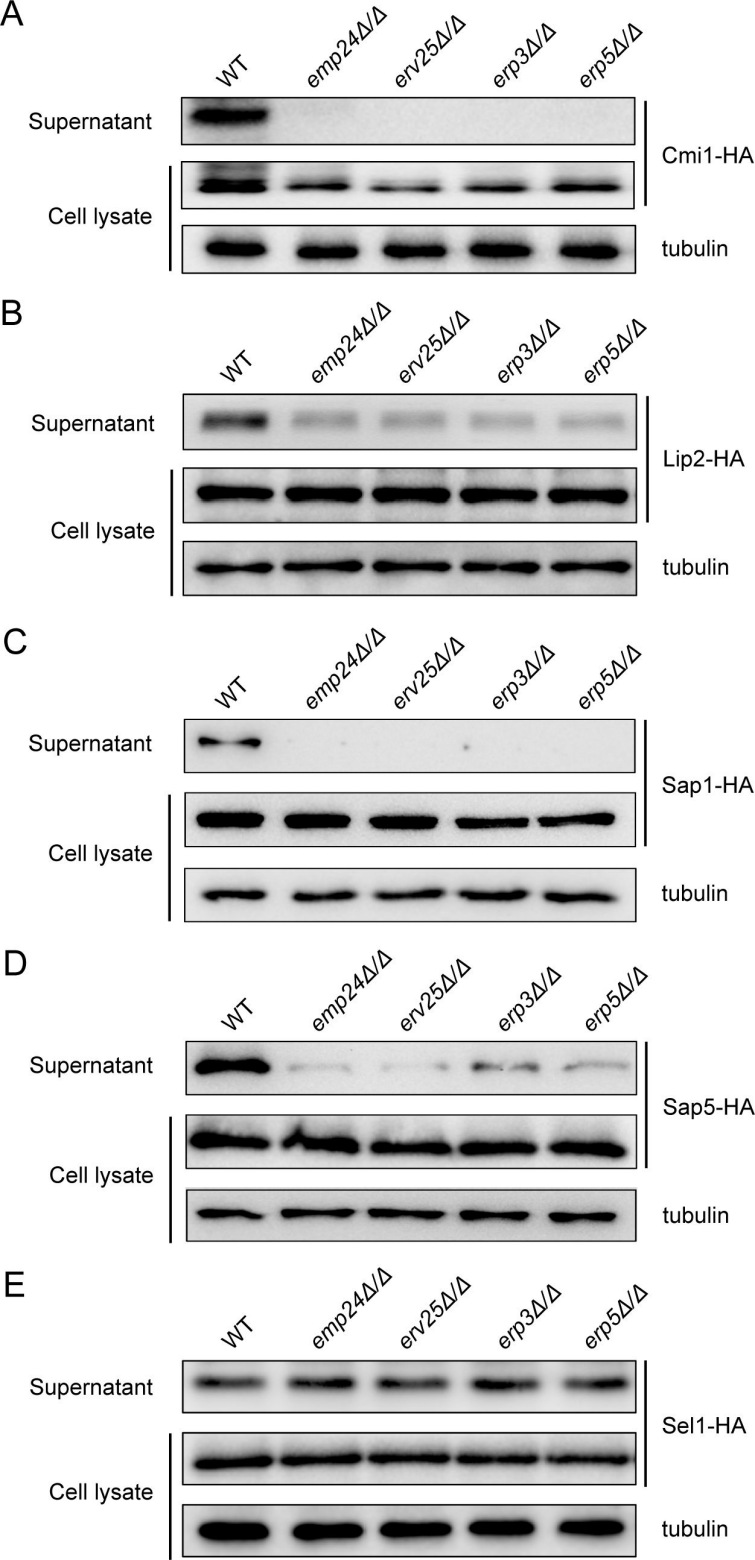
Western blot analysis of cell lysate and supernatant samples. Cells were grown in liquid YPD medium at 30°C. Proteins were extracted from *C. albicans* cells expressing hemagglutinin (HA)-tagged proteins Cmi1 (**A**), Lip2 (**B**), Sap1 (**C**), Sap5 (**D**), or Sel1 (**E**) after incubation for 14 h, and samples were processed to separate intracellular (cell lysate) and extracellular (supernatant) fractions. Representative blots of three independent experiments are shown.

p24 family proteins are known to be involved in protein trafficking along the secretory pathway (20). We therefore examined their potential impact on pathogenicity-related protein secretion. Recently, we identified an effector protein, Cmi1, which can be delivered into host cells to promote both invasive infection and gut colonization by suppressing type I interferon responses in *C. albicans* (11). We first asked whether p24 proteins impact intracellular Cmi1 levels. As shown in Fig. 6A, a reduction in the abundance of Cmi1 protein in cell lysates is observed in *emp24*Δ/Δ, *erv25*Δ/Δ, *erp3*Δ/Δ, and *erp5*Δ/Δ mutant strains compared to that in the WT strain. Remarkably, we could not detect any Cmi1 protein in the supernatant after incubation with the p24 mutant strains, whereas a significant amount of Cmi1 protein was observed in the supernatant following WT *C. albicans* incubation (Fig. 6A). These data indicate that p24 proteins are essential for Cmi1 secretion, and the abundance of Cmi1 protein is partially regulated by p24 proteins.

In addition to Cmi1, we examined four other recently identified functional secreted proteins in *C. albicans*. Lip2 is a secreted lipase essential for virulence in invasive infection (9). Similar to Cmi1, *C. albicans* failed to secrete Lip2 into extracellular space in the absence of p24 proteins (Fig. 6B). However, the intracellular Lip2 protein levels in p24 mutant strains were comparable to that in WT strain (Fig. 6B). The secretion of Sap1 and Sap5, two secreted aspartyl proteinases, exhibited a similar pattern with Lip2 in WT and p24 mutants (Fig. 6C and D). Sel1, a cysteine-rich secreted protein, might function as a pathogen-associated molecular pattern (PAMP) that elicits host proinflammatory response (45). We found that neither protein level nor protein secretion of Sel1 was regulated by p24 proteins (Fig. 6E). Taken together, these results establish that p24 proteins are required for the secretion of certain pathogenicity-related proteins, thereby contributing to the regulation of virulence and gut colonization in *C. albicans*.

## DISCUSSION

The expression levels of the p24 family are upregulated during invasive candidiasis, implicating its critical role in pathogenicity. In this manuscript, we have conducted a loss-of-function approach to investigate the function of p24 proteins in *C. albicans*. In particular, we have generated single deletion mutants affecting each gene of the p24 family. All these mutants showed a strong decrease in virulence compared to WT in a systemic infection model, as well as reduced commensal fitness in competition with WT. In addition, they showed a clear alteration in the structure of cell wall, increased drug susceptibility to fluconazole, and diminished secretion of virulence factors, but no obvious phenotypic alterations under standard growth conditions. Overall, our functional characterization of p24 proteins uncovered broad roles for these proteins in fungal pathogenesis.

Based on sequence homology, the eukaryotic p24 family consists of four subfamilies: α-, β-, γ-, and δ-p24. The genome of *S. cerevisiae* encodes eight proteins of the p24 family, which are distributed among the four conserved subfamilies (46). The p24α and p24γ subfamilies have three members, while the p24β and p24δ subfamilies contain only one single member. The situation is clearly different in *C. albicans*, which seems to have only one member of each subfamily. In addition, a mutant strain lacking all p24 family members in *S. cerevisiae* showed only very mild phenotypic alterations (47). In clear contrast, the knockout of a single p24 family member in *C. albicans* results in attenuated virulence, defective stress responses, and diminished secretion of virulence factors. Thus, despite the striking conservation of p24 family among eukaryotes, their function differs even among yeast species.

The capacity of *C. albicans* to reversibly switch between yeast and hyphal morphologies is the best-known virulence attribute (40, 41, 48). The p24 family is critical for the virulence and commensal fitness of *C. albicans* but dispensable for hyphal morphogenesis. Moreover, the expression of p24 proteins is independent of the yeast-to-hypha transition but is induced by tissue culture conditions and during *in vivo* infection. Our group recently identified Cmi1, which is necessary for virulence and commensal fitness, as an effector protein in *C. albicans* (11). Cmi1 can be translocated into host cells through poorly understood mechanisms and specifically targets TBK1. In the present study, we clearly showed that the secretion of Cmi1 protein was dependent on the presence of all four members of the p24 family. Similar to p24 proteins, Cmi1 is not required for hyphal development, and its expression is not affected by yeast-to-hypha transition (11). These findings suggest that the attenuated virulence and commensal fitness of the p24 mutant strains may result at least partially from impaired Cmi1 secretion.

p24 proteins have long been proposed to function as specific cargo-interacting receptors that facilitate the transport of specific cargoes between the ER and the Golgi (22, 49). Except for Cmi1, the p24 family was also required for the secretion of Lip2, Sap1, and Sap5. By contrast, the p24 family was not required for the secretion of Sel1, an elicitor of the host defense response (45). Unlike other secreted proteins we tested, Sel1 is cysteine-rich, which is thought to stabilize its tertiary structures through disulfide bridges. This may provide an explanation why p24 proteins are dispensable for Sel1 secretion. These results suggest that *C. albicans* evolved multiple mechanisms for the secretion of proteins of different types, and this warrants further investigation. In addition, p24 proteins played an important role in cell wall biosynthesis, as the abundance of chitin and phosphomannan was decreased in p24 mutants. However, other important cell wall components, such as glucan, remained largely unchanged upon deletion of the p24 genes. This may reflect the different responses of p24 mutants to different antifungals. In light of all this evidence, we propose that p24 proteins are involved in modulating pathogenicity in *C. albicans* by mediating efficient and accurate delivery of diverse secretory and membrane proteins.

## MATERIALS AND METHODS

### Media and growth conditions

*C. albicans* was routinely cultured in YPD medium (2% Bacto peptone, 2% glucose, 1% yeast extract) at 30°C. *C. albicans* transformation was performed as described previously (50), and transformants were selected on YPD plates supplemented with 200 μg/mL NAT.

To induce hyphal formation, overnight cultures of *C. albicans* were harvested by centrifugation, washed three times with sterile phosphate-buffered saline (PBS), and resuspended in an equivalent volume of PBS. The cell suspension was then diluted 1:100 into YPD medium supplemented with 10% serum or RPMI-1640 medium and incubated at 37°C. Cells were collected at 4 h for cell morphology analysis.

### Plasmid and strain construction

*C. albicans* strain SC5314 genomic DNA was used as the template for all PCR amplifications of *C. albicans* genes. *C. albicans* strains used in this study are listed in Table S2. The primers used for PCR amplifications are listed in Table S3.

Deletion of the *EMP24*, *ERV25*, *ERP3*, and *ERP5* genes was performed using a CRISPR-Cas9 strategy as follows. The single-guide RNA (sgRNA) was annealed and inserted into the Esp3I site of pV1393 or pV1524 (51). The resulting plasmid was linearized by digestion with KpnI and SacI digestion and transformed into SC5314 along with the corresponding repair template. Successful gene deletions were confirmed through PCR analysis and DNA sequencing.

pBA1-ERV25, pBA1-EMP24, pBA1-ERP3, and pBA1-ERP5 were constructed for the constitutive expression of *ERV25*, *EMP24*, *ERP3*, and *ERP5*, respectively, under the control of the *ADH1* promoter. The full-length coding sequences of *ERV25* (primers 17 and 18), *EMP24* (primers 19 and 20), *ERP3* (primers 21 and 22), and *ERP5* (primers 23 and 24) were obtained by PCR amplification and inserted into the BglII-EcoRV site of pBA1 (52). The resulting plasmids were digested with AscI and integrated into the *ADE2* locus of the *C. albicans* genome.

To express hemagglutinin (HA) fusion proteins under the control of the *ADH1* promoter, the full-length coding sequences for *SEL1* (primers 35 and 36) and *LIP2* (primers 37 and 38) were obtained by PCR amplification. The HA fragment (primers 33 and 34) was obtained from pCPC61 by PCR amplification. The DNA fragments encoding the *SEL1* gene and HA tag were inserted into the BglII-EcoRV site of pBA1 using Gibson assembly method to generate plasmid pBA1-SEL1-HA. Using the same approach, the plasmid pBA1-LIP2-HA expressing Lip2-HA was also constructed.

### Murine model of systemic infection

BALB/c mice were purchased from Beijing Vital River Laboratory Animal Technology Company. Mice were housed in a temperature-constant animal room (22°C) with a reversed dark/light cycle (7:00 a.m. on and 7:00 p.m. off) and 40–70% humidity.

Male BALB/c mice (19–21 g) were intravenously inoculated via the tail vein with 5 × 10^5^ CFU of *C. albicans*. Animals were monitored daily for general health, and survival curves were generated using GraphPad Prism. To quantify fungal burden, 19–21 g male BALB/c mice were inoculated via tail vein injection with 2.5 × 10^5^ CFU of *C. albicans* cells. On day 5 pi, the infected kidneys, spleens, and livers were collected, homogenized, and cultured on agar plates supplemented with streptomycin (100 µg/mL) and ampicillin (50 µg/mL). Colony-forming units were counted following incubation at 30°C.

### Commensal competition experiment in mouse

All animals were singly or doubly housed and provided autoclaved distilled water and autoclaved mouse chow. 19–21 g female BALB/c mice were treated with antibiotic water (streptomycin, 2 mg/mL; penicillin, 0.97 mg/mL) for 3 days and then inoculated via oral gavage with a 1:1 mixture of a NAT-resistant strain and an unmarked strain at 5 × 10^8^ cells/mL, as previously reported (43). The antibiotics water was used throughout the commensal competition experiment. Colonization was tested over time by collecting fresh fecal pellets and plating homogenates on YPD plates containing streptomycin (100 µg/mL) and ampicillin (50 µg/mL) supplemented with or without 200 μg/mL NAT. The CI of the competition experiment has been shown as the proportion of the indicated strain to the total.

### Murine model of mucosal infection

Mucosal infection was assessed as previously described (44) with minor modifications. Male BALB/c mice (19–21 g) received subcutaneous injections of cortisone acetate (3 mg per mouse) in 200 µL PBS containing 0.5% Tween 80 on days −1 and +1 pi. On day 0, mice were anesthetized by intraperitoneal injection of avertin (tribromoethanol: 350 mg/14 mL/kg). A swab soaked in a suspension of 1 × 10^7^ CFU/mL of *C. albicans* cells in sterile saline was placed sublingually for 75 min. Two days pi, infected tongues were collected and homogenized, and serial dilutions were plated on agar plates to quantify fungal burden.

### Infection of macrophages

RAW264.7 cells were cultured in DMEM containing 10% serum and challenged with *C. albicans* at a MOI of 10:1 (macrophage:*Candida*). Non-phagocytosed *Candida* cells were removed by washing with PBS after 1 h of co-incubation. To determine the growth of intracellular *Candida* cells, RAW264.7 cells were lysed with 0.1% Triton X-100 following an additional 5 h of incubation. After resuspension, serial dilution, and plating onto YPD plates, the phagocytized fungal cells were counted. The release of LDH into the culture supernatant was monitored as a measure of RAW264.7 cell damage. Culture supernatants were collected, and the enzymatic activity of LDH was determined using the Cytotoxicity Assay (Promega G1780), according to the manufacturer’s instructions. The absorbance of the blank medium was subtracted as the background value from the observed absorbance of each sample. The relative activity was plotted as % of the control samples.

### Assessment of cell wall components

Log-phase *C. albicans* cells were harvested and washed twice with PBS. Cells were then fixed with 4% paraformaldehyde at 4°C for 30 min, followed by three additional PBS washes. Subsequently, 2 × 10^6^ cells were blocked with PBS containing 2% BSA for 30 min and subjected to a final PBS wash. Surface-exposed β-1,3-glucan was detected using Fc-dectin-1. Fixed cells were incubated with 3 µg/mL Fc-dectin-1 on ice for 60 min, washed three times with PBS, and then incubated with FITC-conjugated secondary antibody (1:200 dilution) on ice for 30 min to quantify surface-exposed β-glucan levels. Total cell wall glucan and chitin content in *C. albicans* was determined using specific fluorescent dyes. For glucan detection, fixed cells were stained with 1% Aniline Blue at room temperature for 30 min. Chitin was visualized using 3.5 µg/mL Calcofluor White (CFW) staining, with fixed cells incubated on ice for 15 min. α-mannose residues were detected using fluorescein-conjugated ConA. Cells were incubated with 25 µg/mL ConA on ice for 30 min for mannan visualization. Phosphomannan incorporation was assessed using Alcian Blue labeling. Log-phase cells were harvested, washed twice with PBS, and resuspended at 1×10^7^ cells/ml in 1 ml of 30 µg/mL Alcian Blue solution. Following a 10-min incubation at room temperature, the mixture was centrifuged, and 200 µL of supernatant, along with standards, was transferred to a 96-well plate for optical density measurement at 620 nm.

### Transmission electron microscopy (TEM)

TEM was performed to examine the ultrastructure of *C. albicans* cell wall. Briefly, *C. albicans* cultures were grown to log phase in liquid YPD medium at 30°C. Cells were harvested by centrifugation at 5,000 × *g* for 5 min at room temperature and washed twice with PBS. For TEM sample preparation, the cell pellets were fixed with 2.5% glutaraldehyde. Following primary fixation, samples were washed three times with PBS and re-fixed with 1% osmium tetroxide. After three additional washes with PBS, the samples were dehydrated through a graded acetone series. The dehydrated samples were infiltrated with Spurr’s resin through a gradient of acetone/resin mixtures and finally embedded in pure Spurr’s resin. Ultrathin sections (70 nm) were prepared using an ultramicrotome and mounted on pioloform-coated copper grids. The sections were examined under a JEOL JEM-1400Plus transmission electron microscope with a digital camera (Gatan Rio 1809). Cell wall thickness was measured using ImageJ software.

### Antifungal susceptibility testing

Drug susceptibility was measured in 96-well plates, and assays were conducted in a total volume of 0.1 mL of YPD medium/well containing different concentrations of antifungal drugs. Cell cultures were prepared with 10^3^ cells in each well. The 96-well plates were incubated in the dark at 30°C for 48 h, after which OD_600_ was measured using a spectrophotometer (BioTek). Data were displayed as heat maps.

### RNA extraction and quantitative PCR expression analysis

To determine the expression of *C. albicans* genes during invasive infection, 19–21 g male BALB/c mice were inoculated with 1 × 10^6^ CFU of *C. albicans* cells via tail vein injection, and infected kidneys were collected at 48 h pi. Total RNA of the infected tissues was extracted using TRIzol reagent (Invitrogen 15596026), following the manufacturer’s instruction. Total RNA from *C. albicans* cells incubated under *in vitro* conditions was purified using the RNAprep Pure Tissue Kit (Tiangen). cDNA was synthesized using the Maxima H Minus cDNA Synthesis Master Mix with dsDNase (Thermo). qRT-PCR analysis was performed using iQ SYBR Green Supermix (Bio-Rad) with gene-specific primers listed in Table S3. Gene expression levels were normalized to *CDC28* mRNA signals.

### Western blotting

The cells were harvested by centrifugation at 4°C, washed, and resuspended in 0.5 mL of lysis buffer (50 mM Tris-HCl, pH 7.5; 150 mM NaCl; 0.1% NP40) supplemented with PMSF and protease inhibitor cocktail (Roche, Indianapolis, IN). Cells were lysed at 4°C using a FastPrep system (FastPrep-24; MP Biomedicals, Solon, OH). Cell lysates were centrifuged for 10 min at 13,000 rpm in a microcentrifuge at 4°C. To detect secreted proteins, the culture supernatant was mixed with SDS-loading buffer and heated to 100°C for 10 min. Equal amounts of proteins were loaded onto each lane, based on OD_280_ measurement, and were separated on 8% SDS-PAGE gel. Separated proteins were electrotransferred to PVDF membrane (Bio-Rad) and blocked with 3% skim milk in PBS containing 0.2% Tween-20 at room temperature for 1 h. Blots were hybridized overnight at 4°C with antibody against the HA epitope (MBL M180-3, 1:5,000 dilution) and tubulin (Proteintech 66240-1-Ig, 1:2,000 dilution). Blots were washed with PBS with 0.1% Tween-20 and subsequently hybridized with FITC-conjugated secondary antibody diluted 1:5,000 in the blocking solution for 60 min at room temperature. Signals were detected using an ECL western blotting kit according to the manufacturer’s instructions (Bio-Rad).

### Quantification and statistical analysis

All experiments were performed with at least three biological repeats, except as indicated in the figure legends, and no statistical method was used to predetermine sample sizes. Analyses were conducted using GraphPad Prism 9.0 software. Results are expressed as the mean ± SD as indicated and analyzed using Student’s *t*-test or log-rank test. *P* values of less than 0.05 were considered statistically significant. ns, no significance; *, *P* < 0.05; **, *P* < 0.01; ***, *P* < 0.001; ****, *P* < 0.0001.

## Data Availability

RNA-Seq data that support the findings of this study have been deposited in the NCBI under BioProject PRJNA1113184. All data reported in this paper will be shared by the corresponding author upon request.

## SUPPLEMENTAL MATERIAL

Supplemental figures and tables msphere.00827-25-s0001.pdf Fig. S1 to S10; Tables S2 and S3.

Table S1 msphere.00827-25-s0002.xlsx *C. albicans* genes that were upregulated by at least 1.5-fold at both 24 and 48 h post-infection.

ASM does not own the copyrights to Supplemental Material that may be linked to, or accessed through, an article. The authors have granted ASM a non-exclusive, world-wide license to publish the Supplemental Material files. Please contact the corresponding author directly for reuse.
