# Supplementary material for: p24 family proteins are critical for cell wall integrity, protein secretion, and virulence in Candida albicans
Source: mSphere. 2026 Jan 20;11(2):e00827-25. doi: 10.1128/msphere.00827-25 (PMC12931272; doi:10.1128/msphere.00827-25)
Supplement: Supplemental figures and tables — Fig. S1 to S10; Tables S2 and S3. [file msphere.00827-25-s0001.pdf]

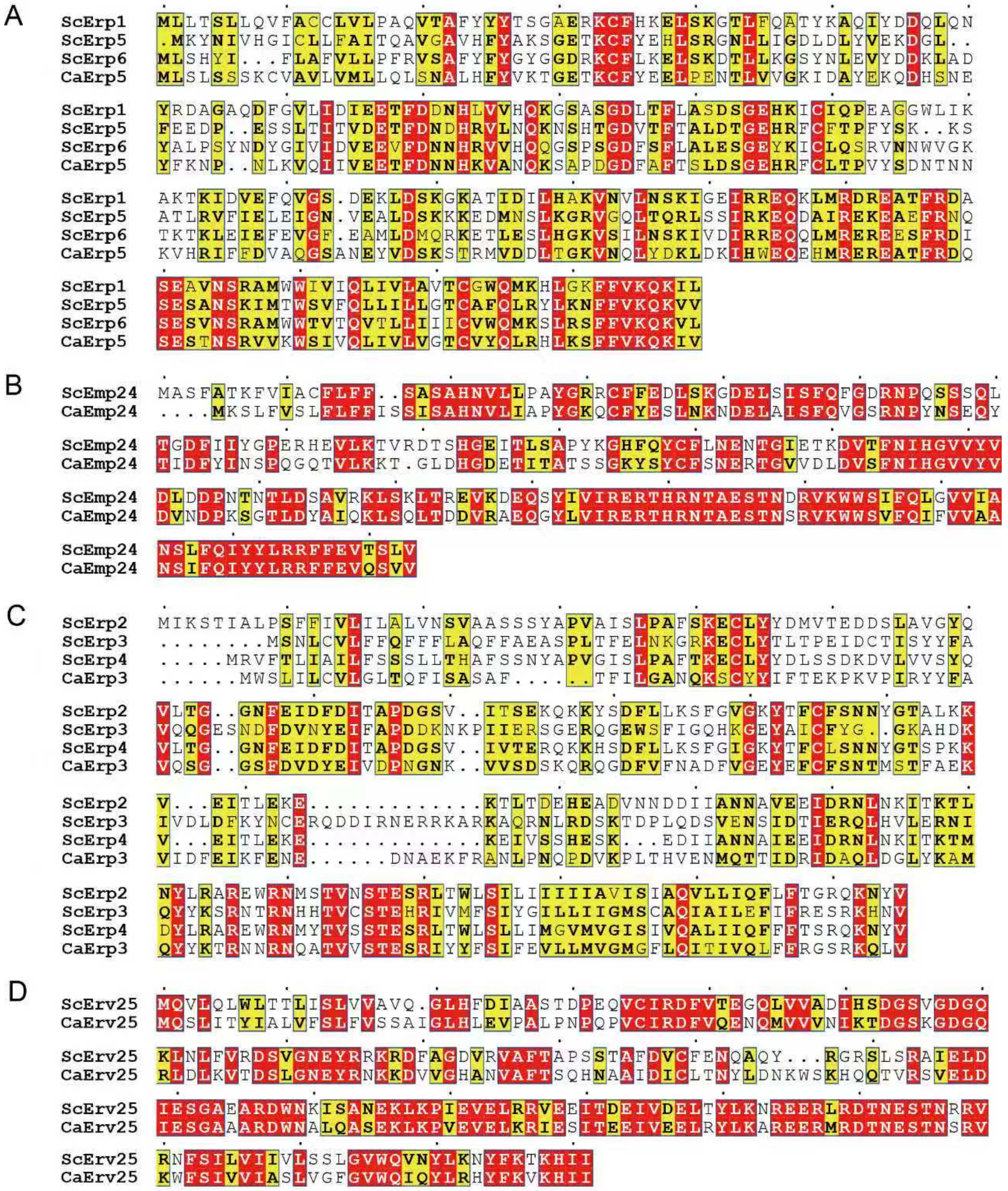

FIG S1 Multiple sequence alignment of p24 subfamily α (A), β (B), γ (C), and δ (D) from *S. cerevisiae* and *C. albicans*. Amino acid sequences are represented using single-letter codes.

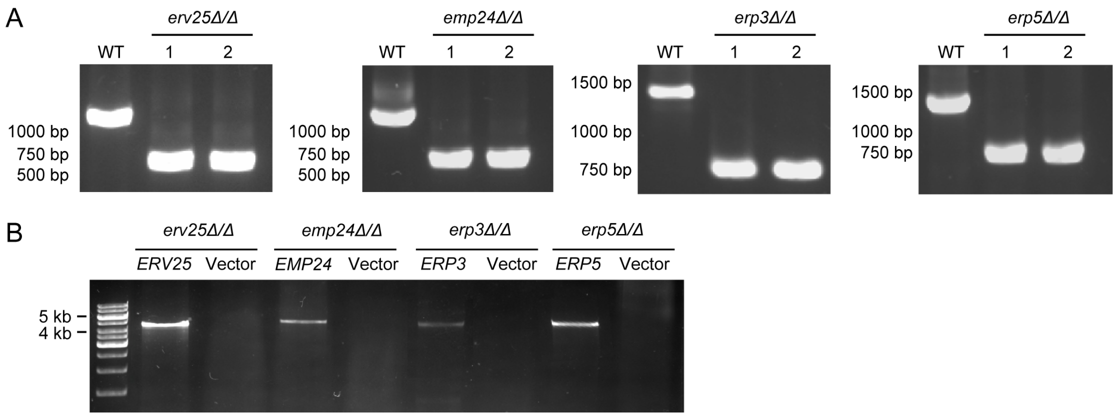

FIG S2 Validation of deletion mutant of p24 genes and complemented strains by PCR. (A) PCR analysis of transformants that carries p24 genes disruption. The products amplified from the region between upstream and downstream of the ORF of p24 genes using genomic DNA of WT and two independent deletion mutants as the template were separated on a gel by electrophoresis. (B) PCR analysis of complemented strains. The products were amplified between *ADE2* integration region and the ORF of p24 genes, which involves a *SAT1* gene and ACT1 promoter.

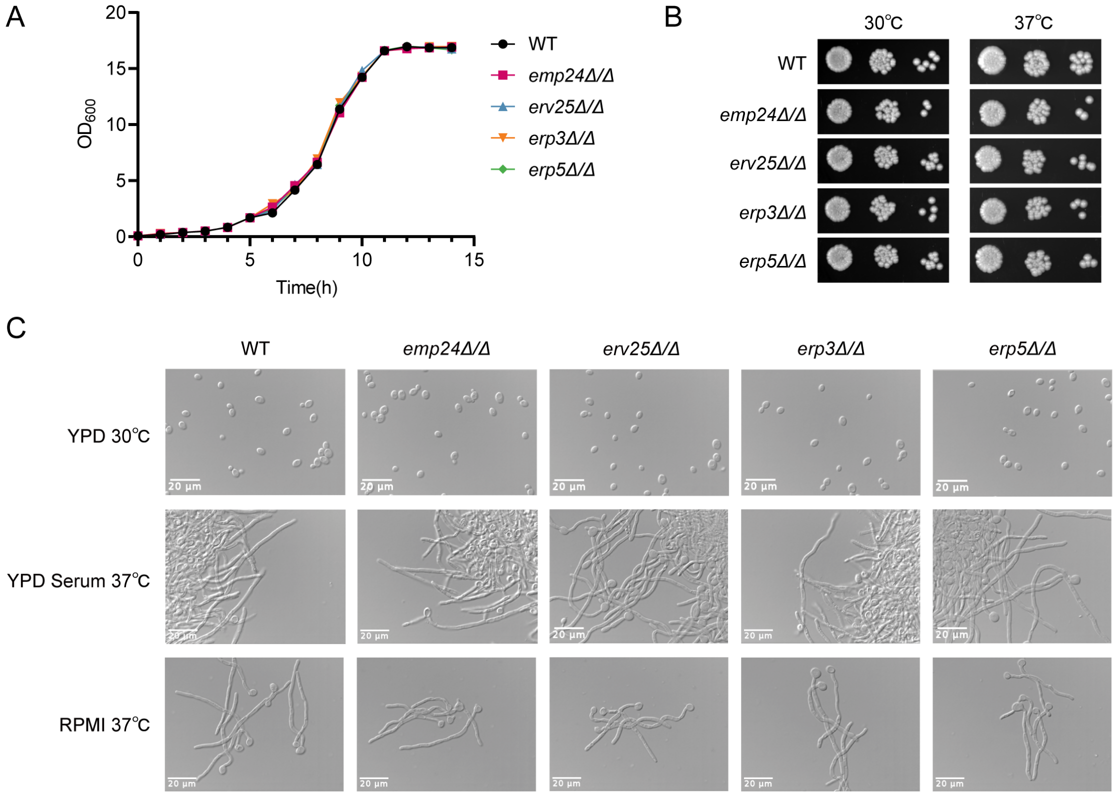

FIG S3 Phenotypic analysis of p24 mutants in *C. albicans*. (A) Growth assays for cells of the WT and indicated mutant strains incubated at 30°C in liquid YPD medium. *C. albicans* cells from overnight cultures were diluted into YPD medium to OD_{600} 0.05 and then tested for growth. (B) Dilutions of the WT and indicated mutant strains were spotted onto YPD solid medium and incubated at 30°C or 37°C. (C) Overnight cultures of the WT and indicated mutant strains were diluted 1:100 into the indicated medium, and cells were collected at 4 h for morphology analysis. Scale bar, 20 μm.

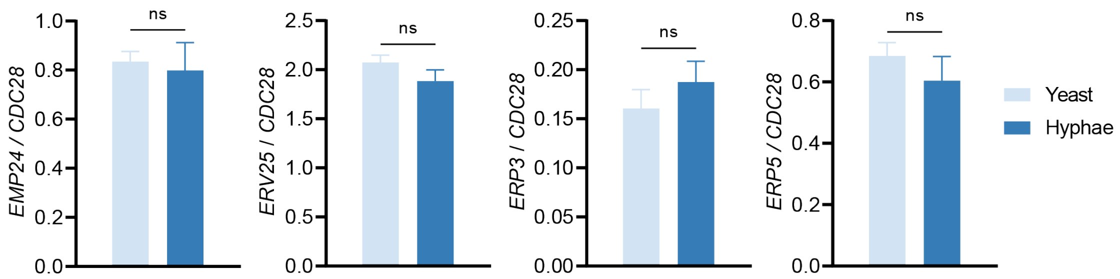

FIG S4 qRT-PCR analysis of the expression of p24 family genes in yeast or hyphae. Overnight cultures were diluted 1:100 and incubated in YPD medium at 30°C for yeast growth or in RPMI-1640 medium at 37°C for hypha induction. Cells were collected at 6 h post-inoculation for RNA extraction. The signals obtained from *CDC28* mRNA were used for normalization. Error bars represent standard deviations from the means of three experiments. Significance was measured with an unpaired t-test in GraphPad Prism. ns, no significance.

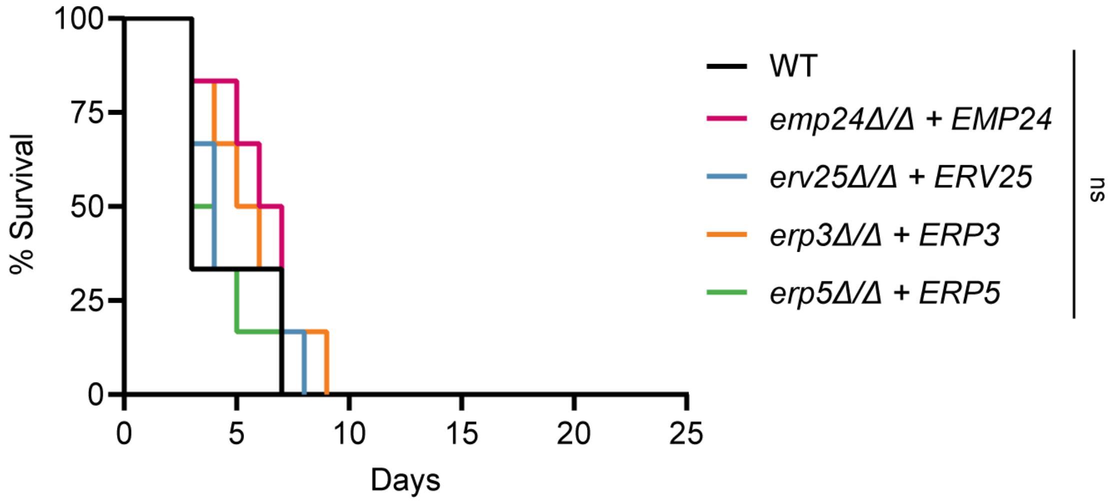

FIG S5 Survival of mice infected with the WT or gene addback strains. For each strain, 6 male BALB/c (19-21 g) mice were injected with 5×10^5 *C. albicans* cells from tail vein. Percentage of survival is indicated in the y-axis. Statistical significance was determined by log-rank test. n = 6 mice. ns, no significance.

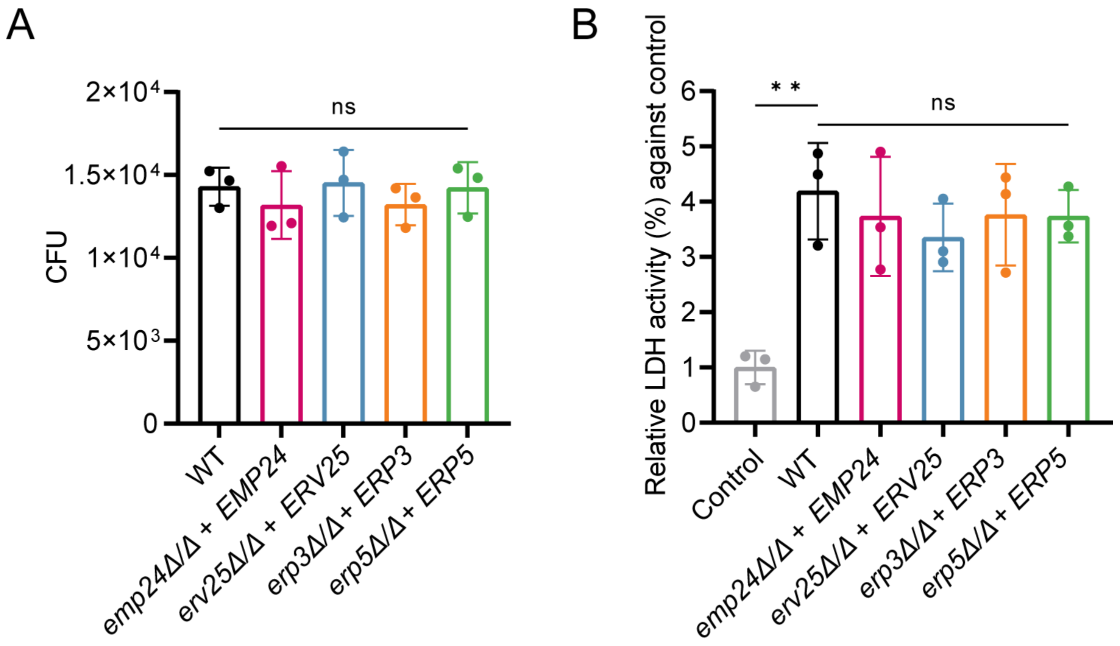

FIG S6 Survival and cytotoxicity assays of the WT or gene addback strains in macrophages. (A) RAW264.7 cells were cultured with the WT strain and indicated strains. Non-phagocytosed *C. albicans* cells were removed by washing with PBS after 1 h, and the CFUs of *C. albicans* in RAW264.7 cells were determined after co-incubation for an additional 5 h. $n = 3$ biologically independent samples. (B) RAW264.7 cell damage following treatment with the WT strain and indicated mutant strains was determined after co-incubation. Relative LDH activity (%) against RAW264.7 cells without *C. albicans* cells was calculated. Mean data ± SD from three independent experiments was plotted. Significance was measured with an unpaired t-test in GraphPad Prism. ns, no significance; **, $p < 0.01$.

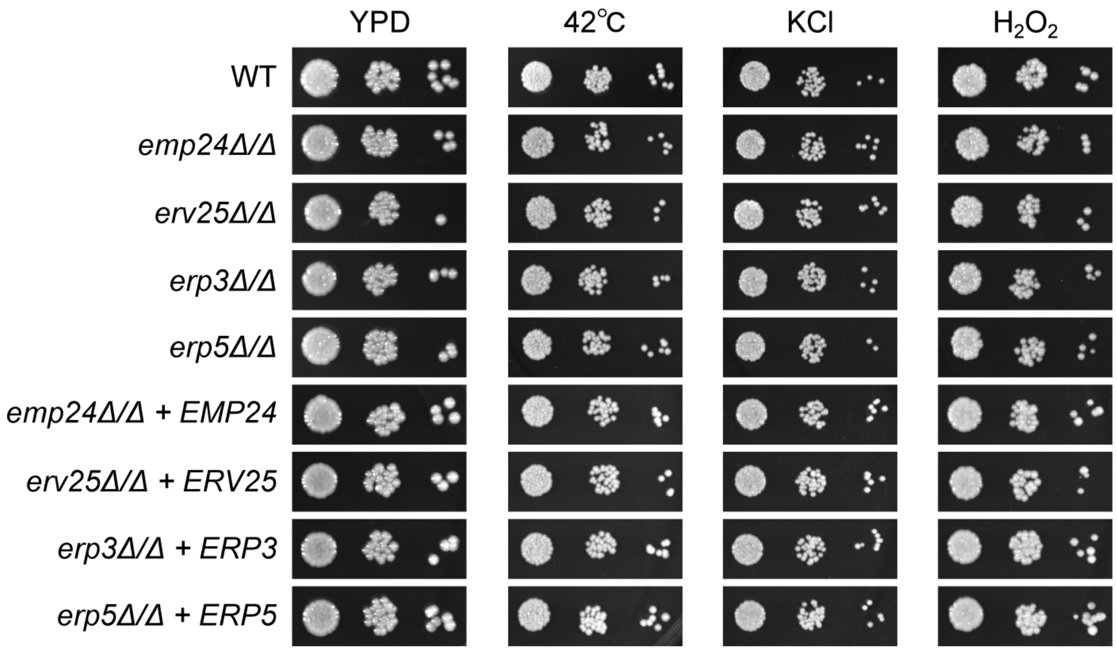

FIG S7 p24 mutants display normal growth in response to KCl, H_2O_2 and high temperature. Cells of WT *C. albicans*, the indicated mutants, and their complemented strains were serially diluted 10-fold and spotted onto YPD solid medium containing 1 M KCl or 5 mM H_2O_2, and incubated at 30℃. Cells were also tested for growth on YPD solid medium at an elevated temperature of 42°C.

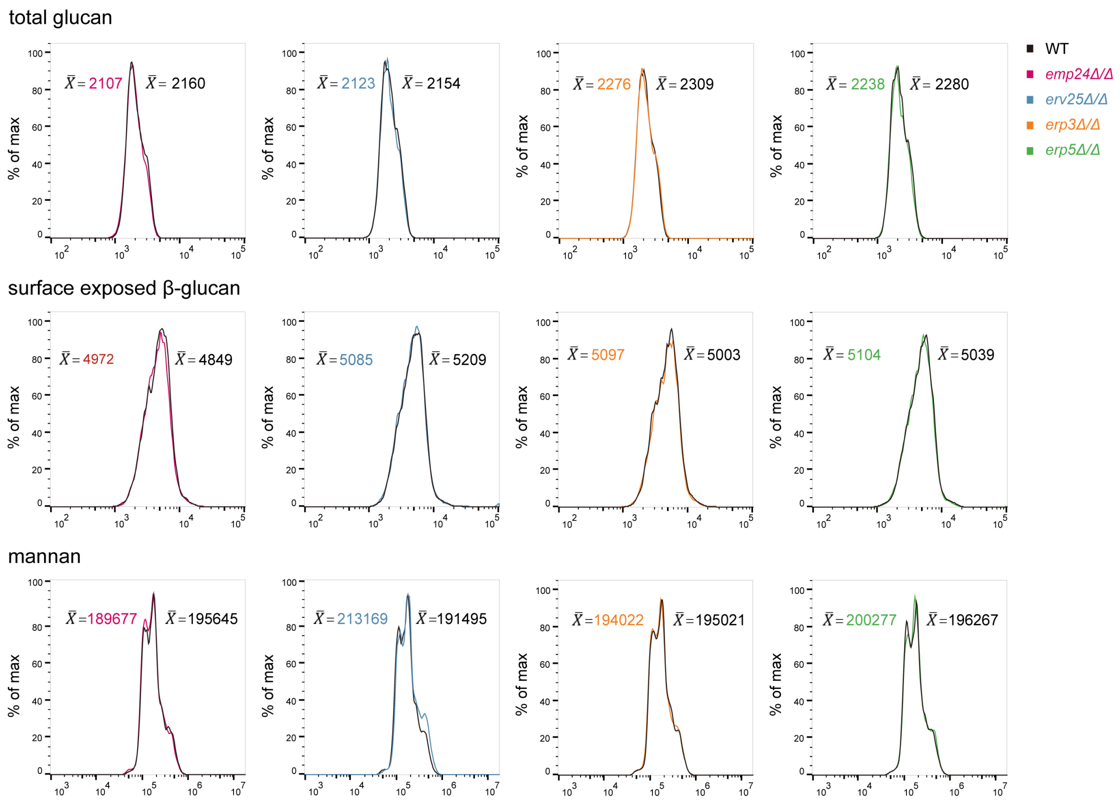

FIG S8 Flow cytometric analysis of cell wall components in the WT and indicated mutant strains. Log-phase *C. albicans* cells were stained with 1% Aniline Blue, 3 µg/mL Fc-Dectin-1, or 25 µg/mL fluorescein-conjugated Concanavalin A to determine total cell wall glucan, surface exposed β-glucan, and cell wall mannan, respectively. Average fluorescence of the population was indicated. Plots are representative of data collected in three independent replicate experiments.

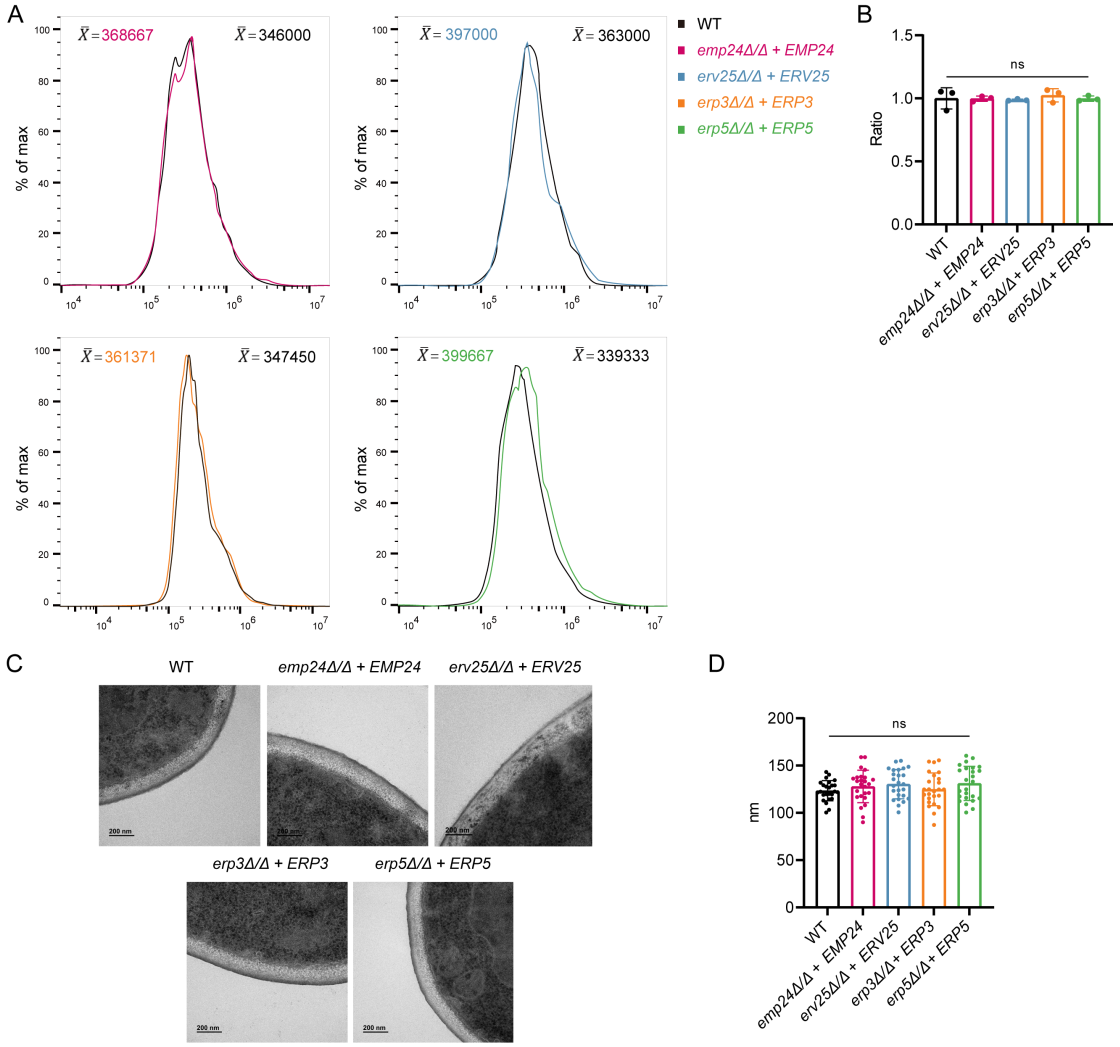

FIG S9 Analysis of cell wall structure of the WT or gene addback strains. (A) Flow cytometric analysis of cell wall chitin content in WT and indicated strains. Log-phase *C. albicans* cells were stained with CFW (3.5 µg/ml) and subjected to fluorescence-activated cell sorting (FACS) analysis. Average fluorescence of the population was indicated. Plots are representative of data collected in three independent replicate experiments. (B) The incorporation of phosphomannan into the fungal cell wall was assessed using Alcian blue staining. Optical density was measured by absorbance at 620 nm following staining with 30 µg/mL Alcian blue, with values averaged from three independent experiments. (C) Transmission Electron Microscope (TEM) images of cell walls from *C. albicans* cells grown in liquid YPD medium at 30°C. Scale bar, 200 nm. (D) Quantification of cell wall thickness based on TEM pictures using ImageJ (n = 25 cells). (B & D) Significance was measured with an unpaired t-test in GraphPad Prism. ns, no significance.

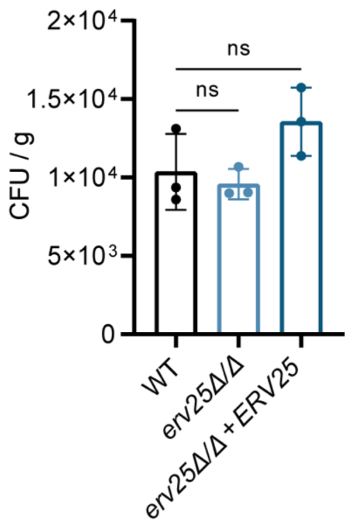

FIG S10 *ERV25* is not required for virulence in mucosal infection model. Fungal burdens recovered from the tongues of mice infected with WT *C. albicans*, *erv25* mutant strain, or *ERV25* gene addback strain after a 2-day oropharyngeal infection. *n* = 3 mice. Significance was measured with an unpaired t-test in GraphPad Prism. ns, no significance.

TABLE S2. ***C. albicans*** **strains used in this study**

Strains	Genotype	Sources
SC5314	Wild type	(1)
YLC60	*ADE2/ade2Δ::SAT1*	(2)
YLC122	*emp24Δ/emp24Δ, ADE2/ade2Δ::FRT*	This study
YLC123	*erv25Δ/erv25Δ, ADE2/ade2Δ::FRT*	This study
YLC124	*erp3Δ/erp3Δ, ADE2/ade2Δ::FRT*	This study
YLC125	*erp5Δ/erp5Δ, ADE2/ade2Δ::FRT*	This study
YLC126	*emp24Δ/emp24Δ, ADE2/ade2Δ::ADH1p-EMP24*	This study
YLC127	*erv25Δ/erv25Δ, ADE2/ade2Δ::ADH1p-ERV25*	This study
YLC128	*erp3Δ/erp3Δ, ADE2/ade2Δ::ADH1p-ERP3*	This study
YLC129	*erp5Δ/erp5Δ, ADE2/ade2Δ::ADH1p-ERP5*	This study
YLC130	*ADE2/ade2Δ::ADH1p-SEL1-3HA*	This study
YLC131	*ADE2/ade2Δ::ADH1p-LIP2-3HA*	This study
YLC132	*ADE2/ade2Δ::ADH1p-CMI1-3HA*	(3)
YLC133	*emp24Δ/emp24Δ, ADE2/ade2Δ:: ADH1p-CMI1-3HA*	This study
YLC134	*erv25Δ/erv25Δ, ADE2/ade2Δ:: ADH1p-CMI1-3HA*	This study
YLC135	*erp3Δ/erp3Δ, ADE2/ade2Δ:: ADH1p-CMI1-3HA*	This study
YLC136	*erp5Δ/erp5Δ, ADE2/ade2Δ:: ADH1p-CMI1-3HA*	This study
YLC137	*emp24Δ/emp24Δ, ADE2/ade2Δ:: ADH1p-SEL1-3HA*	This study
YLC138	*erv25Δ/erv25Δ, ADE2/ade2Δ:: ADH1p- SEL1-3HA*	This study
YLC139	*erp3Δ/erp3Δ, ADE2/ade2Δ:: ADH1p- SEL1-3HA*	This study
YLC140	*erp5Δ/erp5Δ, ADE2/ade2Δ:: ADH1p- SEL1-3HA*	This study
YLC141	*emp24Δ/emp24Δ, ADE2/ade2Δ:: ADH1p-LIP2-3HA*	This study

YLC142	*erv25Δ/erv25Δ, ADE2/ade2Δ*:: *ADH1p- LIP2-3HA*	This study
YLC143	*erp3Δ/erp3Δ, ADE2/ade2Δ*:: *ADH1p- LIP2-3HA*	This study
YLC144	*erp5Δ/erp5Δ, ADE2/ade2Δ*:: *ADH1p- LIP2-3HA*	This study
YLC145	*emp24Δ/emp24Δ, ADE2/ade2Δ*:: *ADH1p-SAP1-3HA*	This study
YLC146	*erv25Δ/erv25Δ, ADE2/ade2Δ*:: *ADH1p- SAP1-3HA*	This study
YLC147	*erp3Δ/erp3Δ, ADE2/ade2Δ*:: *ADH1p- SAP1-3HA*	This study
YLC148	*erp5Δ/erp5Δ, ADE2/ade2Δ*:: *ADH1p- SAP1-3HA*	This study
YLC149	*emp24Δ/emp24Δ, ADE2/ade2Δ*:: *ADH1p-SAP5-3HA*	This study
YLC150	*erv25Δ/erv25Δ, ADE2/ade2Δ*:: *ADH1p- SAP5-3HA*	This study
YLC151	*erp3Δ/erp3Δ, ADE2/ade2Δ*:: *ADH1p- SAP5-3HA*	This study
YLC152	*erp5Δ/erp5Δ, ADE2/ade2Δ*:: *ADH1p- SAP5-3HA*	This study

TABLE S3. Primers used in this study

	Sequence (5'-3')	Purpose and features
1	ATTTGTTGAAAGTTACAGATCTGCTG	Guide *ERV25*
2	AAAACAGCAGATCTGTAACTTTCAAC	
3	CATTAAATAACTCACCTGAAAGTACACAACCATGCAATCATTAATCACACATATTATTT	*ERV25* Repair template
4	GGGAAAGCTTTACCTCAATAGTTTCGACAATAGTAGCTTAAATAATATGTGTGATTAAT	
5	ATTTGCTAACTCCAGAGTCAAATGGG	Guide *EMP24*
6	AAAACCCATTTGACTCTGGAGTTAGC	
7	AGTATTCGCATAGGAACAATCCATCATTAGACTTGAGAACAATTTTACAGAAGGAGATT	*EMP24* Repair template
8	TTGAGACAATGCTAGAATTATACAATGATGTAAACACTAAATCTCCTTCTGTAAAATTG	
9	ATTTGTATTATTTTGCCGTTCAAAGG	Guide *ERP3*
10	AAAACCTTTGAACGGCAAAATAATAC	
11	TTAAACTAGTTTTAACAAGGGATTCATAATCAATCAACAAAATTTCATAAAACCCCTTT	*ERP3* Repair template
12	TATTTATTTCCTTCATTAAACTACATATACACCAACAAAAAAGGGGTTTTATGAAATTT	
13	ATTTGACATTTCTACGTTAAGACCGG	Guide *ERP5*
14	AAAACCGGTCTTAACGTAGAAATGTC	
15	ATTCGTCTTCTACAGACTTTCACAACAACAACAACAACAATACTATATCGCGTATTGCT	*ERP5* Repair template
16	CTATACGACATTAAAAGTAATCAATATAAATCAAACACTAGCAATACGCGATATAGTAT	
17	CAACAAATACAAAAACAAAGATCTATGCAATCATTAATCACATAT	*ERV25* amplification
18	GACGGTATCGATAAGCTTGATATCTTAAATAATATGTTTGACTTT	
19	CAACAAATACAAAAACAAAGATCTATGAAGTCATTATTTGTTTCA	*EMP24* amplification
20	GACGGTATCGATAAGCTTGATATCCTATACAACTGATTGCACTTC	
21	CAACAAATACAAAAACAAAGATCTATGTGGTCATTAATTTTATG	*ERP3* amplification
22	GACGGTATCGATAAGCTTGATATCTTACACCAATTGTTTTCTTG	
23	CAACAAATACAAAAACAAAGATCTATGCTTAGTTTGTCTAGTAG	*ERP5* amplification

24	GACGGTATCGATAAGCTTGATATCTTAAACAATCTTTTGTTTGAC	
25	GCAAGCAAGCGAAAAATT	*ERV25* qRT-PCR
26	ACGCCAAAGCCGACCAAA	
27	GGTCACGTAATCCTTATA	*EMP24* qRT-PCR
28	CATTGCTAAAACAATACG	
29	GTGGATCATTTGATGTTG	*ERP3* qRT-PCR
30	CAGCATTATCTTCATTTTC	
31	GAGAATACTTTGGTTGTG	*ERP5* qRT-PCR
32	CGTCTGGAGCAGACTTTT	
33	AGCGCGCCAATTAGATCC	*HA* amplification
34	GTCGACGGTATCGATAAGCTT	
35	CAACAAATACAAAAACAAAGATCTATGGTTTCATTCAAGTCATTA	*SEL1* amplification
36	CACGGATCTAATTGGCGCGCTTTTAGCACAAGCACCACC	
37	CAACAAATACAAAAACAAAGATCTATGAAAGGTTTGGTTTTCTTA	*LIP2* amplification
38	ACGGATCTAATTGGCGCGCTAATAATACTGCTTACAAGACC	
39	TGGATTAGCTCGAGCATTTG	*CDC28* qRT-PCR
40	CCAACAGACCACATATCTACCC	
41	GATGTGTGGTAAATGAAC	*ERV25* Knockout verification
42	CAAGGATGCGTTATTACA	
43	CTGTCGCCACGCAACTACTA	*EMP24* knockout verification
44	ACGGGCGATACTTAGCGATG	
45	GGTTGTCGATCTTACCAACGC	*ERP3* knockout verification
46	CTGCTTCCAGCAGCAGAG	
47	TCGTTCCATTTTACGCGTTGT	*ERP5* knockout verification
48	AGGAGAGCCTAACCCTCCAG	
49	GAGTTGTGAGGTCTTGGTG	addback verification
50	GGAGAACACCAAGGCAATAT	*ERV25* addback verification
51	GGAGCAATCAACACATTATGAG	*EMP24* addback verification
52	GCTGATGCAGAAATGAATTGAG	*ERP3* addback verification
53	ACGGCAACGCATTTACTAC	*ERP5* addback verification

54	CAAATACAAAAACAAAGATCTATGTTTTTAAAGAAT	
55	CGGATCTAATTGGCGCGCTGGTAAGAGCAGCAATGTTT	Sap1-HA plasmid construction
56	TCAAACATTGCTGCTCTTACCAGCGCGCCAATTAGATCC	
57	GACGGTATCGATAAGCTTGATATCATTTACTGGTGTCCAAGA	
58	CAAATACAAAAACAAAGATCTATGTTCTTGAAAAATATCT	
59	CGGATCTAATTGGCGCGCTATTAATAGCAACAATGT	Sap5-HA plasmid construction
60	CTGACATTGTTGCTATTAATAGCGCGCCAATTAGATCC	
61	GACGGTATCGATAAGCTTGATATCATTTACTGGTGTCCAAGA	

Restriction sites are underlined.

Reference

(1) Fonzi W A, and M Y Irwin. 1993. Isogenic strain construction and gene mapping in Candida albicans. *Genetics* 134,3:717-28.

(2) Yang D, Zhang M, Su C, Dong B, Lu Y. 2023. Candida albicans exploits N-acetylglucosamine as a gut signal to establish the balance between commensalism and pathogenesis. *Nature communications* 14(1), 3796.

(3) Luo G, Zhang J, Wang T, Cui H, Bai Y, Luo J, Zhang J, Zhang M, Di L, Yuan Y, Xiong K, Yu X, Zhang Y, Shen C, Zhu C, Wang Y, Su C, Lu Y. 2024. A human commensal-pathogenic fungus suppresses host immunity via targeting TBK1. *Cell Host Microbe* 32(9):1536-1551.e6.
